# Exergy Analyses of Low-Temperature District Heating Systems With Different Sanitary Hot-Water Boosters

**DOI:** 10.3390/e21040388

**Published:** 2019-04-10

**Authors:** Primož Poredoš, Andrej Kitanovski, Alojz Poredoš

**Affiliations:** 1 Laboratory for Refrigeration and District Energy, Faculty of mechanical Engineering, University of Ljubljana, 1000 Ljubljana, Slovenia; 2 Slovenian Energy Association, 1000 Ljubljana, Slovenia

**Keywords:** exergy efficiency, low-temperature district heating, booster, sanitary hot water

## Abstract

This paper presents an exergy-efficiency analysis of low-temperature district heating systems (DHSs) with different sanitary hot-water (SHW) boosters. The required temperature of the sanitary hot water (SHW) was set to 50 °C. The main objective of this study was to compare the exergy efficiencies of a DHS without a booster to DHSs with three different types of boosters, i.e., electric-, gas-boiler- and heat-pump-based, during the winter and summer seasons. To achieve this, we developed a generalized model for the calculation of the exergy efficiency of a DHS with or without the booster. The results show that during the winter season, for a very low relative share of SHW production, the DHS without the booster exhibits favorable exergy efficiencies compared to the DHSs with boosters. By increasing this share, an intersection point above 45 °C for the supply temperatures, at which the higher exergy efficiency of a DHS with a booster prevails, can be identified. In the summer season the results show that a DHS without a booster at a supply temperature above 70 °C achieves lower exergy efficiencies compared to DHSs with boosters at supply temperatures above 40 °C. The results also show that ultra-low supply and return temperatures should be avoided for the DHSs with boosters, due to higher rates of entropy generation.

## 1. Introduction

District heating is a system for distributing heat from a central source to a large number of users for individual, multi-family residential and commercial heating requirements and industrial processes. Possible sources of heat include heat-only boiler stations, where the heat is produced by burning fossil fuels or renewables, such as wood biomass or municipal waste, or combined heat-and-power plants, where these energy sources can be used more efficiently [1]. The large amounts of by-product heat from cogeneration can only be used in a district heating system. The basic concept of a district heating system is based on hot water flowing through a network of pipes, where heat is carried to substations and from where it is distributed to end users. Over the years, district heating systems have proven to be one of the most cost-effective heating solutions [2].

The Energy Efficiency Directive 2012/27/EU [3] recognizes district heating as one of the key technologies to meet the energy-efficiency targets of the directive, “Member States are required to develop an efficient district heating and cooling infrastructure to accommodate the development of high-efficiency cogeneration and the use of heating and cooling from waste heat and renewable energy sources”. Additionally, in accordance with Implementing the Energy Efficiency Directive—Commission Guidance, 2013, Promotion of energy efficiency in heating and cooling [4] “Member States must examine cogeneration and district heating and cooling first. These systems are recognized as Best Available Techniques (BATs). Only when, and to the extent that these are not technically or economically feasible, taking into account long-term costs and benefits within a given geographical boundary, should Member States proceed with examining other efficient heating and cooling solutions, such as efficient individual heating and cooling”. All this confirms the need for further research and energy-efficiency improvements in district heating systems.

The most substantial energy-efficiency improvements for district heating can be achieved by lowering the temperature of the system to reduce the heat losses in the network [5]. The minimum supply temperature depends on the type of heating installation employed by the end user. Modern low-temperature systems require low supply temperatures and allow for low return temperatures [6]. Due to there being several positive impacts of lowering the supply and return temperatures of such a district heating system (DHS), especially concerning the energy efficiency, DHSs with low supply temperatures down to 35 °C are undergoing pilot testing [7]. They can be supplied from waste-heat recovery systems or renewables, but although these can provide the low temperatures suitable for low-temperature district heating (LTDH) systems, they are insufficient for sanitary hot-water (SHW) production. To overcome this problem, so-called boosters are increasingly common. These boosters raise the temperature of the heat from a district heating system to the standard range of 50–55 °C required for SHW. The technologies applied are dependent on various factors, with the most common booster types being electric-boiler, gas-boiler, and heat-pump boosters [8,9,10].

An exergy analysis that was used to develop a model for the differentiated pricing of heat from a district heating system was conducted back in 2002 [11]. The findings were used to set the price for heat based on exergy loss, and applied in a district heating system with consumers that require varying temperature levels, for instance 90 °C for space heating and 140 °C for industrial processes. As a result, the price of heat for industrial processes was 24% higher than the price for space heating. This prompted an industrial user to implement measures to lower the required supply temperature to 110 °C, thus cutting the costs of heat and reducing the heat losses throughout the pipeline network by about 15%. In one of his papers, Li et al. [12] outlined his thermal and hydraulic simulation of a district heating system designed to supply 30 single-family homes. The energy and exergy analyses were conducted for temperature levels of 55/25 °C. Li found a significant adverse impact of the thermal by-pass on the consumer side.

Researchers have not only examined ways to improve energy efficiency by lowering district heating flow temperatures, they have also analyzed methods to reduce the energy and exergy requirements of booster heating devices for SHW. Based on measurements, Yang et al. [13] performed an energy analysis on five different substation configurations, which used either in-line electric heaters or heat pumps. The results showed that substations with an in-line heater as a booster heating device had better energy and economic performances than the other substations. Yang et al. [14] also carried out an exergy evaluation of solutions for supplying SHW from a low-temperature district heating system with supply temperatures of 35–65 °C, identifying the best booster solutions for the given temperature levels. Yang found that a decentralized substation system with an instantaneous heat exchanger was the best exergo-economic solution in the case that the district heating temperature is high enough for SHW heating. Ommen et al. [15] investigated the optimal integration of a heat-pump booster in an ultra-low-temperature district heating system. Analyzing two possible heat sources, i.e., combined heat and power (CHP) and a central heat pump, the study was made for an energy-efficient, multi-family residential building where heating and SHW accounted for equal shares of the demand. Ommen found that the energy efficiency of the system as a whole depended on factors including the type of heat source, and was not necessarily higher at lower supply temperatures. Meanwhile, in the specific case of a single-family home, Elmegaard et al. [16] ascertained that the best exergo-economic performance was achieved with a booster heat pump. The results of his research showed that a conventional district heating system delivered the highest exergy efficiency and the lowest space heating and SHW costs. In such a system, no booster is required, as the supply temperature is high enough for SHW.

Most of the analyses focused on specific cases with fixed low-temperature district heating boundary conditions for fixed operational settings. No general exergy analyses of the space heating and SHW supply from a district heating system can be found in the literature. Thus, the main objective of this study is to compare the exergy efficiencies of a DHS without a booster to DHSs with three types of boosters: electric-, gas-boiler-, and heat-pump-based. For this purpose, a generalized model for the exergy efficiency of a DHS with or without a booster has been developed. To sum up, this study provides a general exergy analysis of SHW production, in which the following considerations were made:

- a wide range of supply and return temperatures;
- varying shares of heat loss in the pipeline network—x_loss_;
- varying relative share of SHW demand—x_shw_;
- various booster types.

## 2. Description of the Analyzed Systems 

**Figure 1:**
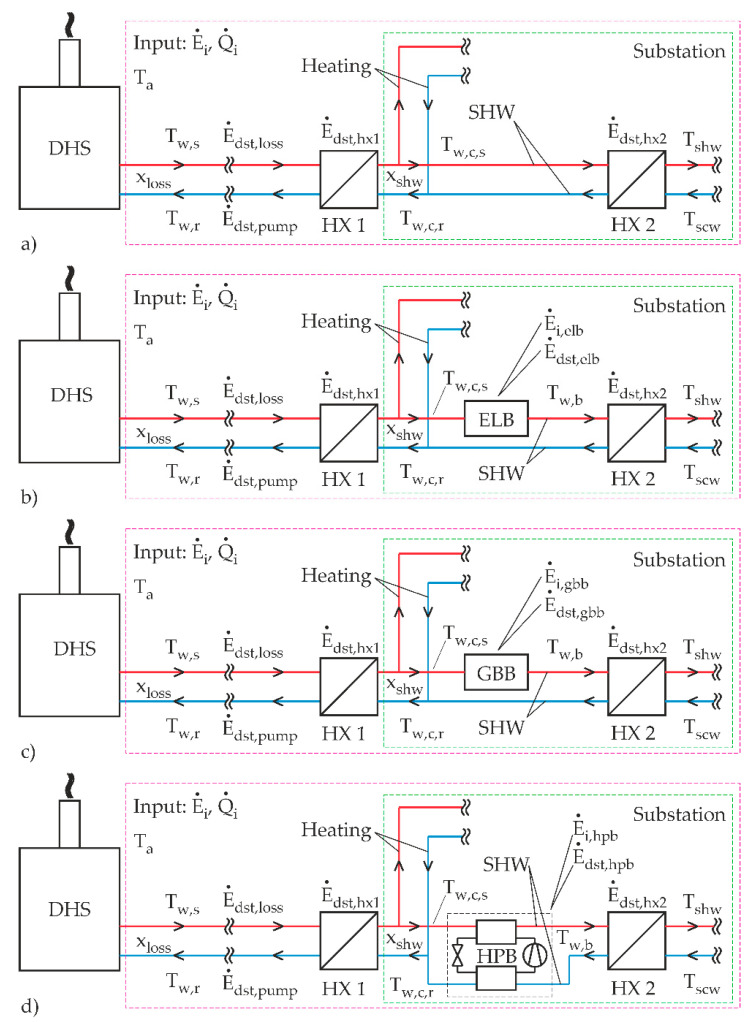
Four cases of SHW production: (**a**) Without a booster; (**b**) using an electric-boiler booster; (**c**) using a gas-boiler booster; (**d**) using a heat-pump booster.

The presented study was performed for four different cases, as shown in Figure 1. The first one, denoted by (a), represents a district heating system (DHS) without a booster, where the supply temperatures (T_w,s_ and T_w,c,s_) are high enough in order to sustain the required temperature of the sanitary hot water (SHW) (T_shw_ = 50 °C). In the second, third, and fourth cases, three different boosters, denoted by (b) electric-boiler-, (c) gas-boiler-, and (d) heat-pump-based, boost the temperature of the SHW to the desired temperature (T_w,b_ = 55 °C). 

Due to the pre-defined temperature difference in the HX 2 (5 °C according to Table 1), T_w,c,s_ and T_w,b_ should be at 55 °C.

The purple square in each case represents a domain that was used for the exergy-efficiency calculations, while the green dashed square describes a substation (secondary side) that is connected to the district heating system (primary side). Each substation has two heat exchangers (HX) with the temperature drops of the supply and return temperatures fixed at 5 °C. All the essential variables that were used during the calculations are shown in Table 1.

## 3. Exergy-analysis model

According to Rant [17], exergy (E) refers to the energy value that has a work potential at the specific state, or in other words, the quality of the energy (EN). The residual energy, denoted as anergy, is defined by the difference:

(1) B = E - EN

The quantity of exergy of the heat can be evaluated by the work output of a Carnot heat engine, which operates between the hot reservoir and the ambient:

(2) $$E_{Q}\;=\;\left(\frac{T_{h}\;{-\;T}_{a}}{T_{h}}\right)Q$$

In order to perform an exergy-based analysis for different SHW productions, the exergy efficiency of each particular system for SHW production, either with or without a booster, was calculated using the following equation:

(3) $$\eta_{\mathrm{ex}}\;=\;1\;-\frac{\sum {\dot{E}}_{\mathrm{dst},m}}{\sum {\dot{E}}_{i,n}}$$

where the values of m and n for the case without a booster are 4 and 1, respectively, and for all three cases with boosters they are 5 and 2, respectively. In order to calculate the efficiency, all the corresponding rates of exergy inputs and destruction terms must be calculated. At the domain input, indicated by the dashed purple square, the rate of exergy input of a district heating system, ${\dot{E}}_{i}$, is calculated using the Equation (2):

(4) $${\dot{E}}_{i}\;=\;\frac{T_{w,m}\;{-\;T}_{a}}{T_{w,m}}{\dot{Q}}_{i}$$

where T_w,m_ represents the mean temperature between the supply (T_w,s_) and the return (T_w,r_) temperatures. The rate of exergy destruction, related to the rate of heat losses between the hot water in the supply and return pipes and the ambient, is calculated using the following equation:

(5) $${\dot{E}}_{\mathrm{dst},\mathrm{loss}}\;={\dot{\;E}}_{i}x_{\mathrm{loss}}$$

The next source of exergy destruction is related to water pumps that require electrical energy, which in fact is pure exergy. If we consider the following relation, as defined by Poredoš et al. [11], which connects the average electric power of the pumps with the hot-water volume flow:

(6) $$P_{\mathrm{el},\mathrm{pump}}\;=\;C\dot{V}$$

where a constant C has the following value of 373 kJ/m^3^ and was acquired by fitting the experimental data. The rate of exergy destruction due to water pumping is in fact anergy, which, by using Equation (1), can be calculated using the following equation:

(7) $${\dot{E}}_{\mathrm{dst},\mathrm{pump}}\;=\;\frac{T_{a}}{T_{w,m}}\left(\frac{C}{\rho_{w,m}c_{w,m}\left(T_{w,s}\;{-\;T}_{w,r}\right)}\right){\dot{Q}}_{i}$$

Since the heat transfer inside the heat exchanger is a source of entropy generation, we could use an expression for the total entropy generation during a Carnot cycle [18]:

(8) $${\Delta \dot{S}}_{\mathrm{gen}}\;=\;-\frac{{\dot{Q}}_{h}}{T_{h}}-\frac{{\dot{Q}}_{c}}{T_{c}}$$

Then the rate of exergy destruction due to heat transfer in heat exchanger 1, denoted as HX 1 (Figure 1), is calculated as follows:

(9) $${\dot{E}}_{\mathrm{dst},\mathrm{hx}1}{\;=\;T}_{a}\Delta {\dot{S}}_{\mathrm{gen}}{\;=\;T}_{a}\left(-\frac{1}{T_{w,m}}+\frac{1}{T_{w,c,m}}\right)\left({1\;-\;x}_{\mathrm{loss}}\right){\dot{Q}}_{i}$$

Please note that the variable T_w,c,m_ represents the mean temperature between the supply and return temperatures on the secondary side (after HX 2). The rate of exergy destruction in the case of a second heat exchanger, denoted as HX 2 (Figure 1), is calculated slightly differently for the scenario with no booster, Figure 1a, by using the equation:

(10) $${\dot{E}}_{\mathrm{dst},\mathrm{hx}2}{\;=\;T}_{a}\left(-\frac{1}{T_{w,c,m}}+\frac{1}{\frac{T_{\mathrm{shw}}{+T}_{\mathrm{scw}}}{2}}\right)\left({1-x}_{\mathrm{loss}}\right){\dot{Q}}_{i}x_{\mathrm{shw}}$$

and scenarios with boosters, Figure 1b–d, by employing the equation:

(11) $${\dot{E}}_{\mathrm{dst},\mathrm{hx}2}{=T}_{a}\left(-\frac{1}{\frac{T_{w,b}{+T}_{w,c,r}}{2}}+\frac{1}{\frac{T_{\mathrm{shw}}{+T}_{\mathrm{scw}}}{2}}\right){K\dot{Q}}_{i}x_{\mathrm{shw}}$$

where K represents the ratio between the booster heating power and the DHS input heating power:

(12) $$K\;=\;\frac{{\dot{Q}}_{b}}{{\dot{Q}}_{i}}$$

The ratio K can be expressed in the following manner:

(13) $$K\;=\;\frac{\rho_{w,b,m}c_{w,b.m}\left(T_{w,b}\;{-\;T}_{w,c,s}\right)}{\rho_{w,c,m}c_{w,c,m}\left(T_{w,c,s}\;{-\;T}_{w,c,r}\right)\left({1\;-\;x}_{\mathrm{loss}}\right)}$$

When using a booster during the SHW production, two additional terms must be taken into consideration: the rate of exergy destruction and the rate of exergy input of each particular booster. The rate of exergy destruction during temperature boosting of the hot water is calculated through the rate of entropy generation:

(14) $${\dot{E}}_{\mathrm{dst},b}{\;=\;T}_{a}{\dot{S}}_{\mathrm{gen},b}$$

where the rate of entropy generation can be expressed as:

(15) $${\dot{S}}_{\mathrm{gen},b}\;=\left(\frac{\ln\frac{T_{w,b}}{T_{w,c,s}}}{\left(T_{w,b}{\;-\;T}_{w,c,s}\right)}\right){K\dot{Q}}_{i}x_{\mathrm{shw}}$$

The rate of exergy input in the case of an electric-boiler booster can be calculated by taking into consideration the following equation:

(16) $${\dot{E}}_{i,\mathrm{elb}}\;=\;\frac{\sum {\dot{E}}_{\mathrm{dst},m}}{1\;-\frac{T_{w,b}\;{-\;T}_{w,c,r}}{T_{w,b}}}$$

By following the second-law efficiency of a heat pump, the rate of exergy input in the case of a heat-pump booster (HPB) is defined as:

(17) $${\dot{E}}_{i,\mathrm{hpb}}=\frac{\sum {\dot{E}}_{\mathrm{dst},m}}{1\;-\;\frac{\mathrm{SPF}\left(T_{w,b}\;{-\;T}_{w,c,r}\right)}{T_{w,b}}}$$

where SPF represents the seasonal performance factor of a water-water heat pump. The SPF is the ratio between the generated heat and the consumed electrical energy. The SPF values were determined by performing simulations using the test reference year (TRY) for Ljubljana [19]. For more information regarding the determination of the SPF, please refer to an article by Poredoš et al. [10]. Table 2 shows the relation between the SPF of a water-water heat pump and the return temperatures at the consumer base (secondary side).

The rate of exergy input of the gas-boiler booster is defined by using the exergy efficiency of a gas-fired boiler with the value of 0.328, as proposed by Terhan et al. [20]:

(18) $${\dot{E}}_{i,\mathrm{gbb}}\;=\;\frac{\sum {\dot{E}}_{\mathrm{dst},m}}{{1\;-\;\eta }_{\mathrm{ex},\mathrm{gas}}}$$

## 4. Results and Discussion

In this section, the results of the performed exergy analysis for four different cases of SHW production with and without boosters are presented below.

### 4.1. Case A: Heating Season with a Variable Share of SHW Production

The first batch of comparisons for four different cases of SHW production is shown for the scenario of a heating season with a variable share of SHW production. A variable share of SHW production makes sense due to the different annual energy consumptions for heating purposes of a particular building. For instance, when analyzing passive houses, the ratio between the energy used for SHW and the energy used for heating purposes is above 50%, while for old houses that require retrofitting, this ratio is below 50%. 

**Figure 2:**
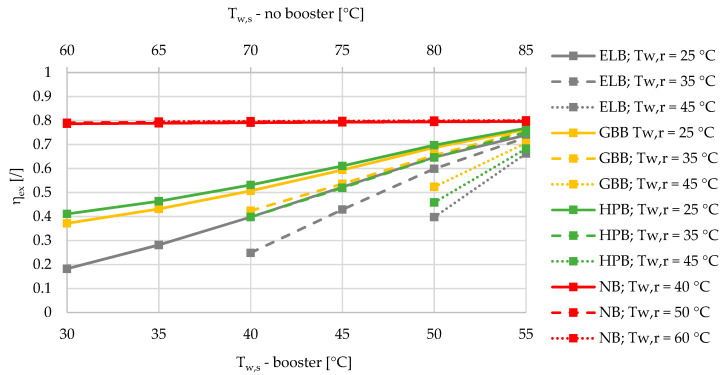
Comparison of the exergy efficiencies of the electric-boiler, gas-boiler, and heat-pump boosters versus the case without a booster for the relative share of SHW production at 10% during the winter.

**Figure 3:**
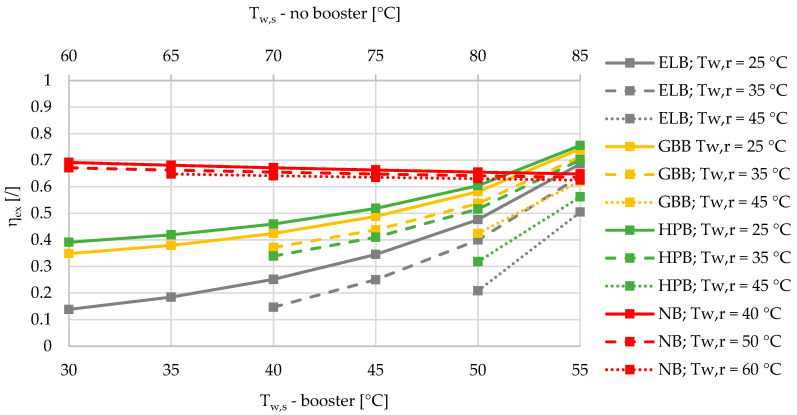
Comparison of the exergy efficiencies of the electric-boiler, gas-boiler, and heat-pump boosters versus the case without a booster for the relative share of SHW production at 50% during the winter.

**Figure 4:**
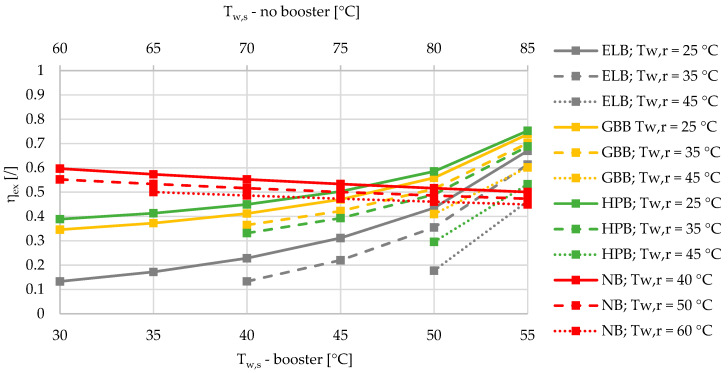
Comparison of the exergy efficiencies of the electric-boiler, gas-boiler, and heat-pump boosters versus the case without a booster for the relative share of SHW production at 90% during the winter.

**Figure 5:**
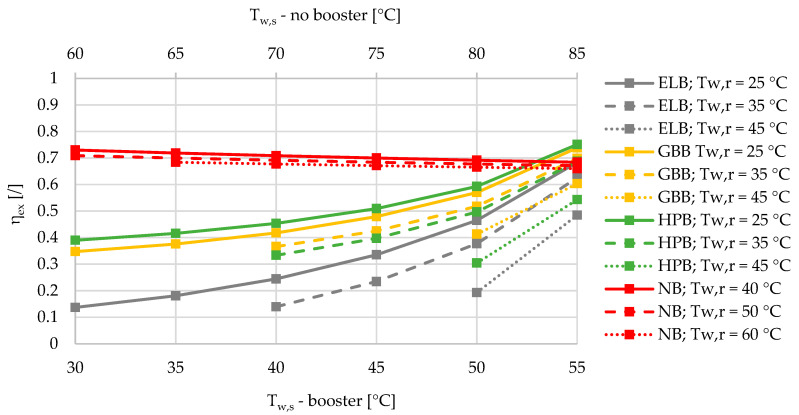
Comparison of the exergy efficiencies of the electric-boiler, gas-boiler, and heat-pump boosters versus the case without a booster for the relative share of heat losses at 5% during the winter.

**Figure 6:**
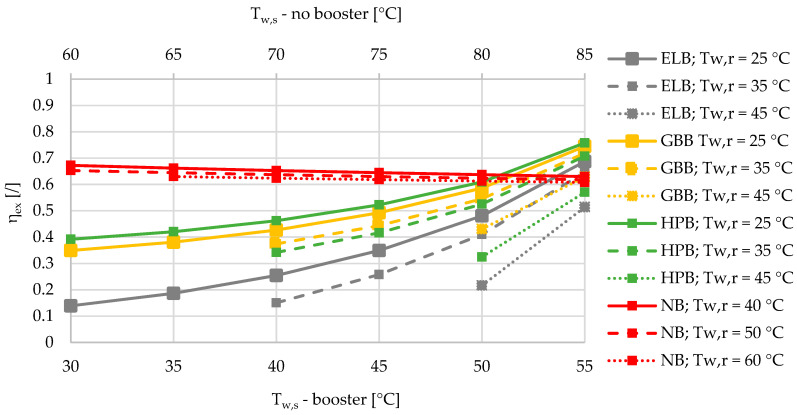
Comparison of the exergy efficiencies of the electric-boiler, gas-boiler, and heat-pump booster versus the case without a booster for the relative share of heat losses at 12.5% during the winter.

**Figure 7:**
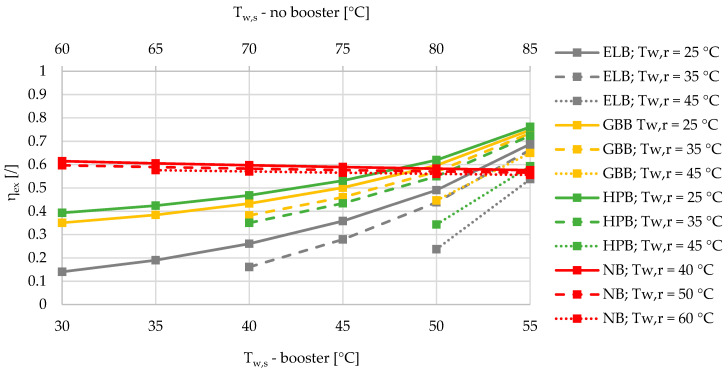
Comparison of the exergy efficiencies of the electric-boiler, gas-boiler, and heat-pump booster versus the case without a booster for the relative share of heat losses at 20% during the winter.

**Figure 8:**
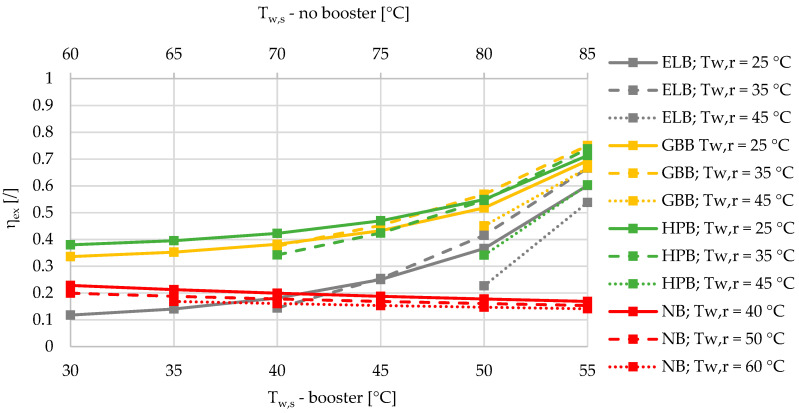
Comparison of the exergy efficiencies of the electric-boiler, gas-boiler, and heat-pump booster versus the case without a booster during the summer.

The mean ambient temperature in the heating season between October and April was calculated based on the TRY for Ljubljana (N 46 3′ 23′’, E 14 30′ 29′’) with the value of 6.0 °C. The heat losses of the DHS were fixed at 10%. Please note that in Figure 2, Figure 3, Figure 4, Figure 5, Figure 6, Figure 7 and Figure 8, there are two x axes for the sake of a comparison between all four different SHW production cases. The SHW production without a booster includes higher supply temperatures of a DHS compared to the SHW production with a booster. 

Based on the results of an exergy-based analysis among DHSs with boosters for three different relative shares of SHW at 10%, 50%, and 90% (Figure 2, Figure 3 and Figure 4), we can observe that for all the supply temperatures of the DHS, the HPB has the highest exergy efficiency in the case of the lowest return temperature at 25 °C. The gas-boiler booster (GBB) is the second in terms of exergy efficiency across the whole range of supply temperatures at the return temperature of 25 °C, while the electric-boiler booster (ELB) has the highest rate of entropy generation. For all three boosters it is also evident that the overall exergy efficiency increases when lowering the return temperatures. When comparing the DHS without a booster to the DHS with all three types of boosters for a 10% of SHW share, the DHS without a booster has an advantage over the DHSs with boosters from the exergy point of view. However, increasing the relative share of SHW production leads to a point that represents an intersection for which the SHW production with a booster is more exergy efficient. At 50% and 90% of the relative shares of SHW production, the intersection points are between 50 and 55 °C for all three boosters, and between 45 and 50 °C supply temperatures for the heat-pump-based and gas-boiler-based boosters, respectively.

A very interesting observation can be made from Figure 2, Figure 3 and Figure 4 regarding the exergy efficiency in the whole supply-temperature range. The SHW production without a booster exhibits an increasing exergy efficiency at 10% of SHW relative share. The main reason for this is the increased supply temperatures, which yield lower mass flow rates due to the higher temperature differences, and consequently less exergy is destroyed during the hot-water pumping. However, because of increasing temperature differences between the SHW, fixed at 50 °C and the supply temperature, a larger amount of exergy is destroyed during the supply-temperature increase.

### 4.2. Case B: Heating Season with Variable Share of Heat Losses

In another case, depicted below in Figure 5, Figure 6 and Figure 7, we see the exergy efficiency during the heating season for three different relative shares of heat losses at 5%, 12.5%, and 20%. Please note that the relative share of the SHW production was fixed at 50%. While the exergy efficiency of the DHS with a booster is slightly affected by the increased heat losses, the DHS without a booster exhibits significantly lower exergy efficiencies. At a relative share of the heat losses at 5%, the intersection point where the exergy efficiencies of the HPB, ELB, and GBB at return temperatures of 25 °C and 35 °C are comparable to those of the DHS without a booster is between 50 and 55 °C of the supply temperatures. For increased heat losses at 12.5% and 20%, the intersection point exhibits a shift towards lower supply temperatures (Figure 6 and Figure 7).

### 4.3. Case C: Summer Season—Sanitary Hot-Water Production

The last case represents a scenario during the summer season from May until September with a mean ambient temperature of 19.2 °C, as calculated from the TRY for Ljubljana. The relative share of heat losses was set to 50%, while the relative share of SHW production was set to 100%. The former parameter has a realistic value due to the fact that in the summer time the relative heat losses are much higher than in the winter time. This is because the absolute amount of heat supplied for the SHW is much lower compared to total amount of heat for heating and SHW purposes in the winter time, while the heat losses of buried DHS pipelines remain nearly the same. Based on the results from Figure 8, the DHS without a booster represents a significantly lower exergy efficiency compared to the DHSs based on the ELB and GBB. Compared to the ELB, the DHS without a booster shows an advantage only for supply temperatures lower than 70 °C and supply temperatures of the DHS with the ELB below 40 °C. 

There is a very interesting additional observation regarding the exergy-efficiency values at the proposed return temperatures of the DHS with all three types of boosters. It seems that the optimal return temperature is 35 °C during the summer season, compared to 25 °C, which has a lower exergy efficiency above 40 °C (GBB), 45 °C (ELB), and 50 °C (HPB) for the supply temperature.

## 5. Conclusions

This paper presents an exergy-efficiency analysis of a low-temperature DHS with different SHW boosters. The main objective of this study was to compare the exergy efficiencies of a DHS without a booster to DHSs with three types of boosters: electric-, gas-boiler-, and heat-pump-based. For this purpose, a generalized model for the exergy efficiency of a DHS with or without a booster has been developed. The final highlights of the exergy analyses for a low-temperature DHS with different boosters for SHW production, based on the presented results, are the following:

- A low-temperature DHS with boosters should avoid extremely low supply and return temperatures, since compared to a DHS without a booster, the former systems exhibit a higher rate of entropy destruction, which lowers the exergy efficiency.
- In the future, low-temperature DHSs with boosters will be more suitable compared to a DHS without boosters when the relative share of the SHW production will be close to 100%.
- The higher return temperatures of the DHSs with boosters are more susceptible to lower exergy efficiencies compared to the DHS without a booster. In the future, special attention should be given to lowering the return temperatures by utilizing different approaches.
- Places with a higher average ambient temperature should opt for retrofitting the high-temperature DHS by upgrading the DHS to a low-temperature system with boosters.
- For the summer operation of a DHS with a booster, an optimization approach should be used in order to determine the most appropriate return temperatures.

### Addendum

The presented article aims to address one of the fundamental questions regarding the eligibility of different SHW boosters in sustainable energy supply chains that are founded on LTDH systems. The developed generalized exergy model, based on basic thermodynamic relations, allows us to determine the optimal temperature regime of a DHS for the SHW production, which ensures the minimal use of exergy. The optimal (low) temperature level of the system water ensures lower energy and also lower exergy losses in the environment, while the optimal choice of booster means the minimal use of energy for sanitary purposes. By selecting the appropriate booster through the utilization of modern technologies, the minimal use of primary energy, and thus minimal emissions for the selected primary energy source, can be achieved. The long-term impact of such decisions led to a sustainable energy chain from production, through distribution, and then to end-use of the energy.

If, for instance, the supply and return temperatures of the DHS are lowered from 85/60 °C to 55/25 °C, the exergy efficiencies of the DHS with and without a booster are comparable for all four cases (Figure 3). However, for the case of the Slovenian energy market, with specific emission factors for electricity production and heat produced by DHS, HP, and natural gas with values of 0.3585, 0.2328, 0.0980 and 0.2020 kgCO_2_/kWh, the reduction in CO_2_ emissions can be up to 20%. This number is valid for the case with a share of SHW production equal to 50%, a share of heat loss equal to 10%, and an ambient temperature of 6 °C. Compared to the DHS at 85/60 °C without the booster with a specific emission factor of 232.8 kgCO_2_/MWh, the DHS with ELB, HPB, and GBB shows the following increases/decreases (also in percentage, %) of specific emission factors: +11.8 (+5.1%), −36.2 (−15.6%), and −17.1 (−7.3%) kgCO_2_/MWh. If the annual amount of heat distributed by the DHS in Slovenia is 2332 GWh, the annual increase/reduction in CO_2_ for the cases of the DHS with ELB, HPB, and GBB at 55/25 °C compared to the DHS without the booster at 85/60 °C, with annual emissions of 271,445 tCO_2_, are +13,750, −42,250, and −19,890 tCO_2_.

## Figures and Tables

**Table 1:** Values of the essential variables used during the calculations.

Variable	Value(s)
T_a_	6 °C (winter), 19.2 °C (summer)
x_loss_	5%, 10%, 12.5, 20 % (winter), 50% (summer)
x_shw_	10%, 50%, 90%
ΔT_hx,1_	5 °C
ΔT_hx,2_	5 °C
T_w,b_	55 °C
T_shw_	50 °C
T_scw_	15 °C
W/o booster	
T_w,s_	60, 65, 70, 75, 80, 85 °C
T_w,r_	40, 50, 60 °C
Booster	
T_w,s_	30, 35, 40, 45, 50, 55 °C
T_w,r_	25, 35, 45 °C

**Table 2:** SPF of a water-water heat pump at different return temperatures (evaporation temperatures) on the consumer side (secondary side). Please note that the temperature of the hot water produced by a HPB is fixed at 55 °C.

T_w,c,r_ [°C]	25	35	45
SPF [/]	3.66	4.00	4.34

## Abbreviations

BAT	best available techniques
CHP	combined heat and power
DHS	district heating system
ELB	electric-boiler booster
HX	heat exchanger
GBB	gas-boiler booster
HPB	heat-pump booster
LTDH	low-temperature district heating
SHW	sanitary hot-water
SPF	seasonal performance factor
TRY	test reference year

## Nomenclature

B	Anergy (J)
c	specific heat (J/kg·K)
C	constant (kJ/m^3^)
E	exergy (J)
$\dot{E}$	rate of exergy (W)
EN	Energy (J)
K	ratio (/)
P	electric power (W)
Q	Heat (J)
$\dot{Q}$	heating power (W)
$\dot{S}$	rate of entropy (J/K·s)
SPF	seasonal performance factor (/)
T	temperature (K)
$\dot{V}$	volume flow (m^3^/s)
x	share (/)

## Greek Letters

η	efficiency (/)
ρ	density (kg/m^3^)

## Subscripts

a	ambient
b	booster
c	consumer; cold reservoir
dst	destruction
el	electric
elb	electric-boiler booster
ex	exergy
gas	gas
gbb	gas-boiler booster
gen	generated
h	hot reservoir
hpb	heat-pump booster
hx	heat exchanger
i	input
loss	losses
m	constant, mean
n	constant
pump	pumping
r	return
s	supply
scw	sanitary cold-water
shw	sanitary hot-water
tot	total
w	water
